# Recent Trends on Applications of Electronics Pervading the Industry, Environment and Society

**DOI:** 10.3390/s20247295

**Published:** 2020-12-18

**Authors:** Sergio Saponara, Alessandro De Gloria, Francesco Bellotti

**Affiliations:** 1 DII (Dipartimento di Ingegneria della Informazione), University of Pisa, via G. Caruso 16, 56122 Pisa, Italy; 2 DITEN (Electrical, Electronics and Telecommunication Engineering and Naval Architecture Department), University of Genoa, via Opera Pia 11/a, 16145 Genoa, Italy; adg@elios.unige.it (A.D.G.); franz@elios.unige.it (F.B.)

This Editorial analyzes the manuscripts accepted, after a careful peer-reviewed process, for the Special Issue “Applications in Electronics Pervading Industry, Environment and Society—Sensing Systems and Pervasive Intelligence” of the Sensors MDPI journal. The Special Issue was co-organized by the University of Pisa (Professor Sergio Saponara at the Department of Information Engineering) and University of Genoa (Professors Alessandro de Gloria and Francesco Bellotti at the Department of Electrical, Electronics and Telecommunication Engineering and Naval Architecture) in Italy. Most of the papers were selected as the best papers of the 2019 edition of the “Applications in Electronics Pervading Industry, Environment and Society” (Applepies) Conference that was held in Pisa in September 2019. All these papers were significantly enhanced with novel experimental results, as we show in the following.

The selected papers give an overview of the trends in research and development activities about the pervasive application of electronics to the industry, environment and society. The focus of the papers is on cyber physical systems (CPS) with research proposals for new sensor acquisition and ADC (analog-to-digital converter) methods, high-speed communication systems, cybersecurity and data processing, including emerging machine-learning techniques.

For each paper, the physical implementation aspects are always discussed, as well as the trade-off to be found between functional performances and hardware costs is exhaustively analyzed.

The Special Issue is characterized by 13 original research papers [1,2,3,4,5,6,7,8,9,10,11,12,13] that we briefly introduce in the following.

The first paper [1] is entitled “A Portable Support Attitude Sensing System for Accurate Attitude Estimation of Hydraulic Support Based on Unscented Kalman Filter”, written by Xuliang Lu, Zhongbin Wang, Chao Tan, Haifeng Yan, Lei Si and Dong Wei from the School of Mechatronic Engineering, China University of Mining and Technology, Daxue Road, Xuzhou 221116, China.

The paper proposes the design of a support attitude sensing system composed of an inertial measurement unit (IMU) with MEMS (microelectromechanical system) sensors. In the classis attitude, a control system’s yaw angle estimation with magnetometers is disturbed by the perturbed magnetic field generated by a coal rock structure and by high-power equipment. On the other hand, roll and pitch angles are often estimated using a MEMS gyroscope and accelerometer, and the accuracy is not reliable with time, usually due to long-term bias instability problems. In order to eliminate the measurement error of the sensors and to obtain an accurate attitude estimation, the paper proposed the use of an unscented Kalman filter based on quaternion, according to the characteristics of complementation of the magnetometer, accelerometer and gyroscope. Then, the gradient descent algorithm is used to optimize the key parameter of the unscented Kalman filter—namely, processing the noise covariance—to improve the accuracy of the attitude calculation. An industrial application shows that the average measurement error of the yaw angle is less than 2° and that of the pitch angle and roll angle are less than 1°, which proves the efficiency and feasibility of the proposed cyber physical system.

The second paper [2] is entitled “Analysis and Comparison of Rad-Hard Ring and LC-Tank Controlled Oscillators in 65 nm for SpaceFibre Applications”, written by D. Monda et al. from the University of Pisa and by INFN (the Italian National Institute for Nuclear Physics).

This work presents a comparison between two voltage-controlled oscillators (VCOs) designed in a commercial 1.2-V 65-nm CMOS (complementary metal oxide semiconductor) technology to address the needs of SpaceFibre, a recent standard for high-speed communications in space applications. The first architecture based on a ring oscillator (RO) was designed using three current mode logic (CML) stages connected in a loop, while the second one was based on an LC-tank resonator. This analysis aimed to choose a VCO architecture able to be integrated into a rad-hard phase-locked loop to meet the specifications of the SpaceFibre protocol, supporting frequencies up to 6.25 GHz. The paper presents the full custom schematic and the layout designs. The single-event effect simulation results, performed according to an IMEC (Interuniversity MicroElectronics Center, Belgium) model with a double-exponential current pulses generator, are also discussed. The performance of the RO-VCO are quite attractive in terms of technology scaling and reduced area occupation. However, the RO-VCO solutions suffer from larger frequency spreading due to process variations and due to operations in harsh space conditions. On the contrary, the LC-VCO solution is characterized by a lower sensitivity to PVT (process–voltage–temperature) variations. Hence, the LC-VCO architecture is the one selected to fulfill the specifications of the new SpaceFibre aerospace standard.

The third paper [3] is entitled “Analysis and Design of Integrated Blocks for a 6.25 GHz SpaceFibre PLL” and is written by M. Mestice at al., with authors from the University of Pisa and by INFN (the Italian National Institute for Nuclear Physics).

Additionally, this paper refers to the SpaceFibre standard for high-speed communication in space applications and presents the design of the key blocks for a phase-locked loop (PLL) to generate the clock reference up to 6.25 Gbps: triple-modular redundancy phase/frequency detector, charge pump and a passive loop filter. Modeling and simulation activities were carried out in the ADS (Advanced Design System) RadioFrequency environment and in the Cadence Virtuoso environment. The results achieved proved that the PLL can be fully integrated on-chip in a commercial 1.2-V 65-nm CMOS technology with an area size dominated by the passive loop filter. Both system-level and layout-level rad-hard techniques were proposed. The results achieved showed that a compact (0.09 mm^2^) and low-power (about 10 mW) dead zone-free and rad-hard PLL can be obtained with a phase noise below −80 dBc/Hz @ 1 MHz and targeting the 6.25-Gbps maximum data rate of the SpaceFibre standard.

The fourth paper [4] is entitled “Machine Learning on Mainstream Microcontrollers” and is authored by F. Sakr at al. from University of Genoa.

This work addresses the emerging problem of implementing machine-learning (ML) techniques in edge devices. More in detail, the paper introduces the edge-learning machine (ELM), a machine-learning framework for edge devices. The goal is managing the ML training phase on a desktop computer, while the inference phase is implemented on a STM32 (STMicroelectronics 32-bit) microcontroller. By using a platform-independent C language, the paper deals with several supervised ML algorithms (a support vector machine with a linear kernel, k-nearest neighbors and decision tree) and exploits the capability of the STM X-Cube AI to implement artificial neural networks (ANNs) on STM32 Nucleo boards. Multiple datasets are considered for classifications and regression. The results of the research work prove that the edge platforms reach the same performance score of a desktop computing platform with a similar time latency. To support the community of developers and makers, the ELM framework is released as an open source.

The fifth paper [5] deals with cybersecurity. It is entitled “Cryptographically Secure Pseudo-Random Number Generator IP-Core Based on SHA2 Algorithm” by L. Baldanzi et al., a group of authors from the University of Pisa.

The adoption of advanced sensors and systems for autonomous driving, combined with an increased connectivity of vehicles, robots and drones, is increasing the importance of embedding security features in computing and communication platforms. To this aim, RNG (random number generation) has a crucial role in ensuring the robustness of the security chain. RNG is a key technology to generate the encryption keys to be used for ciphers. Hence, any weakness in the key generation process will lead to leaks of information and will increase the probability to breach even the strongest cipher. More in detail, the paper shows the architecture of a CSPRNG (cryptographically secure pseudo-random number generator) macrocell that was validated by using the official Statistical Test Suite of the NIST (National Institute for Standard and Technology) to assess the degree of randomness of the numbers generated. The proposed CSPRNG macrocell was characterized by both FPGA (field-programmable gate array) and ASIC (application-specific integrated circuit) standard-cell technologies.

The sixth paper [6] is entitled “Data Processing and Information Classification—An In-Memory Approach” and is authored by M. Andrighetti et al. from Politecnico di Torino.

In the Internet of Things (IoT) era, an enormous amount of data, generated by billions of electronic devices full of sensors that constantly acquire data, must be processed and classified. A classic approach is transferring these data to servers that elaborate them remotely in the cloud. This approach is energy-inefficient (there is a huge battery drain due to the high amount of information to be transferred) and is affected by latency problems in safety-critical time-sensitive applications. Data may be processed locally in edge devices, near the sensor itself, but this solution requires a high-performance computation (HPC) and memory capability that is often missing in mobile microprocessors and microcontrollers. To address these issues, the paper presents a PIM (processing-in-memory) approach where new memories are designed to elaborate the data inside them, overcoming the well-known “memory wall” issue. More in detail, the work, with reference to a bitmap indexing case study, presents a hardware accelerator designed in CMOS technology around the PIM approach. The hardware accelerator is capable of implementing the bitmap indexing algorithm and can also be reconfigured to implement other tasks. The achieved results show that the PIM approach allows to process and classify huge amounts of data locally, with a very low power consumption.

The seventh paper [7] is entitled “Digital Circuit for Seamless Resampling ADC Output Streams” and is authored by M. D’Arco et al. from the Federico II University of Naples.

This work first presents DSP (digital signal processing) techniques to change the sampling rate of digital storage oscilloscopes (DSOs) by means of digital resampling approaches. Then, it proposes a new digital circuit to be included in the acquisition channel of a DSO, between the internal analog-to-digital converter (ADC) and the acquisition memory, that allows the user to select any sampling rate lower than the maximum one with fine resolution. The new circuit exploits both digital-filtering techniques with dynamically generated coefficients and ad-hoc memory management strategies. For the circuit, both FPGA and ASIC implementations are evaluated.

The eighth paper [8] is entitled “Embedded Bio-Mimetic System for Functional Electrical Stimulation Controlled by Event-Driven sEMG” and is authored by F. Rossi et al., a group of authors from Politecnico di Torino.

This paper deals with an assistive technology application and, particularly, with the surface electromyographic (sEMG) signal for controlling the functional electrical stimulation (FES) therapy, a technique that is being widely accepted as an active rehabilitation method for the restoration of neuromuscular disorders. To this aim, the paper proposes an embedded implementation of the average threshold crossing (ATC)-FES control system. The system was characterized and validated by analyzing the computing core and memory usage in different operating conditions, as well as measuring the system latency. Experimental results on a testing population of 11 subjects was also carried out.

The ninth paper [9] is entitled “Fast Approximations of Activation Functions in Deep Neural Networks when using Posit Arithmetic” and is authored by M. Cococcioni et al., a group from the University of Pisa and the company MMI srl.

The paper addresses the problem of an arithmetic format to achieve real-time computing for deep neural networks (DNNs). Overcoming the limits of a classic IEEE 754 standard floating-point representation, the paper presents a new numerical format called Posits. While waiting for the widespread availability of hardware-native Posit Processing Units, the paper shows that it is possible to exploit the Posit representation and the currently available arithmetic-logic unit (ALU) in every microprocessor to speed up DNNs by manipulating the low-level bit string representations of Posits. To this aim, the paper presents a new class of Posit operators called L1 operators, which consists of fast and approximated versions of existing arithmetic operations or functions, such as the hyperbolic tangent (TANH) and the extended linear unit (ELU), while being adopted as activation functions in DNNs. The achieved results show that using either 64-bit ARM processors or x86 Intel 10-bit Posits can represent an exact replacement for 32-bit floats, while 8-bit Posits could be an interesting alternative to 32-bit floats, since their performances are a bit lower, but their high-speed and-low storage properties are very appealing. This size reduction will lead to a lower bandwidth demand and more cache-friendly codes. Moreover, with only 8- or 10-bit Posit operations, they can be tabulated in a very efficient way.

The tenth paper [10] is entitled “Steerable-Discrete-Cosine-Transform (SDCT): Hardware Implementation and Performance Analysis” and is from R. Peloso et al., a group of authors from Politecnico di Torino. 

This paper deals with the hardware acceleration of new, efficient video compression methods and, particularly, the steerable discrete cosine transform (SDCT) that were proposed to exploit directional DCT using the basis of having different orientation angles. With respect to classic solutions, the SDCT leads to a sparser representation and, hence, to an improved compression efficiency. The hardware accelerator for SDCT processing proposed in this paper is able to work at about 200 MHz with a throughput of 3G sample/s and can support an 8k UHD (ultra-high definition) format at 60 frames per second.

The eleventh paper [11] is entitled “Distillation of an End-to-End Oracle for Face Verification and Recognition Sensors” and is authored by F. Guzzi et al., a group of authors from the University of Trieste, the Elettra Sincrotrone Trieste S.C.p.A and the Abdus Salam International Centre for Theoretical Physics.

This paper deals with face recognition functions that are important for many applications, including security systems, inclusion devices and others. In this work, a distillation technique is applied to a complex model to enable fast recognition on low-complex hardware face recognition sensors. The proposed biometric systems are examined for the two problems of face verification and face recognition in an open set by using training/testing methodologies and datasets.

The twelfth paper [12] is entitled “A Model-Based Design Floating-Point Accumulator. Case of Study: FPGA Implementation of a Support Vector Machine Kernel Function” and is authored by M. Bassoli et al., a group of authors from the University of Parma.

This paper presents a novel model-based floating-point accumulation circuit (relying on the state-of-the-art delayed buffering algorithm) to accelerate ML functions, such as the kernel function of support vector machines. The proposed model was implemented in a Simulink environment and then implemented, by means of an HDL (hardware description language) design, in FPGA technology. The simulation results showed that it has a better performance in terms of speed and occupied area when compared to other solutions. To better evaluate its figure, a practical case of a polynomial kernel function was considered.

Finally, paper thirteen [13], “Managing Big Data for Addressing Research Questions in a Collaborative Project on Automated Driving Impact Assessment”, is authored by Bellotti et al., an international group of researchers collaborating in the L3Pilot project, which is the piloting of Society of Automotive Engineers (SAE) level 3 automated vehicle functions.

The paper presents the development of a set of tools for a big data management process involving several project actors (vehicle manufacturers, research institutions, suppliers and developers), with different perspectives and requirements. In order to implement a reference methodology, the authors highlight the importance of (i) a common data format to process all the source data coming from proprietary sources, (ii) a measurement-oriented application programming interface (API) for storing and retrieving data and of a tool to synthetize meaningful data from the original, proprietary vehicular time series.
